# Clinical pattern of failure after a durable response to immune check inhibitors in non-small cell lung cancer patients

**DOI:** 10.1038/s41598-021-81666-x

**Published:** 2021-01-28

**Authors:** Ja Yoon Heo, Shin Hye Yoo, Koung Jin Suh, Se Hyun Kim, Yu Jung Kim, Chan-Young Ock, Miso Kim, Bhumsuk Keam, Tae Min Kim, Dong-Wan Kim, Dae Seog Heo, Jong Seok Lee

**Affiliations:** 1 Department of Internal Medicine, Seoul National University Hospital, Seoul, Republic of Korea; 2 Department of Internal Medicine, Bundang Hospital, Seoul National University College of Medicine, 82, Gumi-ro 173 beon-gil, Bundang-Gu, Seongnam, Gyeonggi-do 13620 Republic of Korea; 3 Cancer Research Institute, Seoul National University, Seoul, Republic of Korea; 4 Present Address: Department of Internal Medicine, National Health Insurance Service Ilsan Hospital, Goyang, Republic of Korea

**Keywords:** Cancer, Tumour immunology

## Abstract

Although immune checkpoint inhibitors (ICIs) can induce durable responses in non-small-cell lung cancer (NSCLC) patients, a significant proportion of responders still experience progressive disease after a period of response. Limited data are available on the clinical patterns of acquired resistance (AR) to ICIs. Clinical and radiologic data from 125 NSCLC patients treated with anti-PD-1 or PD-L1 antibodies between 2011 and 2018 at two tertiary academic institutions were retrospectively reviewed. Overall, 63 (50.4%) patients experienced AR after ICI treatment in a median of 10.7 months. Among the 13 patients with a partial response with ICI, 12 (32.4%) had only lymph node progression. Most patients (n = 52, 82.5%) had one or two sites with progression (oligo-progression). The median overall survival (OS) after progression was significantly longer in the extrathoracic group than in the thoracic and liver progression groups (30.2 months [95% confidence interval (CI), 13.4 to not reached (NR)], 11.7 months [95% CI, 9.5–21.1], and 5.4 months [95% CI, 2.6-NR], respectively, *P* < 0.001). Patients with oligo-progression had significantly longer OS after AR than did the multi-progression patients (18.9 months [95% CI, 10.6-NR] vs. 8.8 months [95% CI, 5.7-NR], *P* = 0.04). No significant difference in progression-free survival was observed between the subsequent chemotherapy and the ICI after AR groups (*P* = 0.723). Patients with AR after ICI treatment had a unique progression pattern with oligo-progression and high rates of progression only in the lymph nodes. Local treatment and/or continuation of ICIs beyond AR might be an effective option.

## Introduction

Immune checkpoint inhibitors (ICIs), including anti- programmed death 1 (PD-1) or programmed death ligand 1 (PD-L1) antibodies, represent a breakthrough in the treatment of non-small-cell lung cancer (NSCLC). Previous studies have shown that high levels of PD-L1 expression on tumors and/or immune cells by immunohistochemistry are associated with improved clinical efficacy when compared with low levels or negative PD-L1^1–6^. In patients with pretreated advanced NSCLC, ICI monotherapy resulted in durable clinical responses lasting more than 6 months and enabled patients to achieve 5-year overall survival (OS) without subsequent therapy and disease progression^6–8^. However, a significant proportion of long-term responders still experience progressive disease after a period of response time.

Acquired resistance (AR) refers to a clinical scenario in which cancer initially responds to immunotherapy, but it continues to grow and metastasize to other organs after a period of time. In the epidermal growth factor receptor (EGFR) mutant NSCLC, it is well-known that acquired mutations, including T790M, confer AR to tumors after EGFR tyrosine kinase inhibitor (TKI) treatment^9^. However, among patients with ICI treatment, more efforts are needed to elucidate which patients will experience AR and how tumor evolution or changes in the immune system lead to AR development. Defects in interferon-receptor signaling and antigen presentation pathways, tumor-associated neoantigen loss, alternate immune checkpoints, and alterations in the tumor microenvironment are believed to be involved in AR mechanisms^10–12^.

Recently, Gettinger et al.^13^ presented an impressive single-institution case series of 26 NSCLC patients who experienced AR of the PD-1 blockade. The authors reported the lymph node (LN) as the most common site of AR; their study included 11 cases with only LN progression. The majority of the included patients (88%) showed progression in one or two sites of disease. Overall, 15 (58%) patients received local therapy to sites of AR, while 11 (42%) patients continued immunotherapy beyond progression^14^. To date, clinical data regarding subsequent treatment strategies and survival according to progression patterns after AR to ICI in NSCLC patients is sparse, especially for those with durable responses.

Therefore, in this study, we conducted a retrospective analysis to investigate the clinical patterns of AR after a durable response to ICIs in NSCLC patients. Additionally, we determined the implications for subsequent therapy in these patients.

## Results

### Patient characteristics

Of the 125 patients with a durable clinical benefit more than 6 months, 63 (50.4%) patients with AR to ICIs were identified. The baseline clinicopathological characteristics of these patients are summarized in Table 1. The majority (n = 47, 74.6%) of the patients were male, and just over half of the patients were pathologically diagnosed with adenocarcinoma (n = 35, 55.5%). The majority of patients received ICI as first or second-line treatment for metastatic NSCLC (n = 47, 74.6%); 21 (33.3%) and 26 (41.3%) patients received it as first and second-line treatment, respectively. The study included 15 (23.8%) patients with driver mutations. Fifty-two (82.5%) patients received monotherapy, while 11 (17.5%) patients received combination therapy, including five (7.9%) patients with PD-1 and CTLA-4 inhibitors, five (7.9%) patients with PD-L1 and CTLA-4 inhibitors, and one (1.6%) patient with a PD-1 inhibitor and chemotherapy. The median duration of follow-up was 18 months (range, 8–60) and 23 (36.5%) patients were alive at the time of data analysis in December 2018.

**Table 1:** Patient characteristics.

	N = 63
**Age (years)**	
Median (range)	64 (34–84)
**Sex**	
Male	47 (74.6)
Female	16 (25.4)
**Ethnicity**	
Asian	63 (100.0)
**Smoking**	
Never smoked	21 (33.3)
Current or ex-smoker	42 (66.7)
**Pathology**	
Adenocarcinoma	35 (55.5)
Poorly differentiated	4 (6.3)
Sarcomatoid	3 (4.8)
Large cell neuroendocrine	2 (3.2)
Squamous	19 (30.2)
**Driver mutation**	
EGFR	11 (17.4)
KRAS	2 (3.2)
ALK	1 (1.6)
ROS1	1 (1.6)
Not identified	48 (76.2)
**ICI treatment line**	
1	21 (33.3)
2	26 (41.3)
≥ 3	16 (25.4)
**ICI treatment**	
PD-1 inhibitor	36 (57.1)
PD-L1 inhibitor	16 (25.4)
PD-1 inhibitor + CTLA-4 inhibitor	5 (7.9)
PD-L1 inhibitor + CTLA-4 inhibitor	5 (7.9)
PD-1 inhibitor + chemotherapy	1 (1.6)
**PD-L1 expression**	
≥ 50%	18 (28.6)
1–49%	10 (15.9)
0%	11 (17.5)
Not available	24 (38.1)
**Best objective response**	
Stable disease	26 (41.3)
Partial response	37 (58.7)
Complete response	0 (0)

*EGFR* epidermal growth factor receptor, *ALK* anaplastic lymphoma kinase, *PD-1* programmed death 1, *PD-L1* programmed death ligand 1, *CTLA-4* Cytotoxic T-Lymphocyte Associated Protein 4.

*Data are presented as the median (range) or number (%).

### Clinical patterns of ICI failure

**Figure 1:**
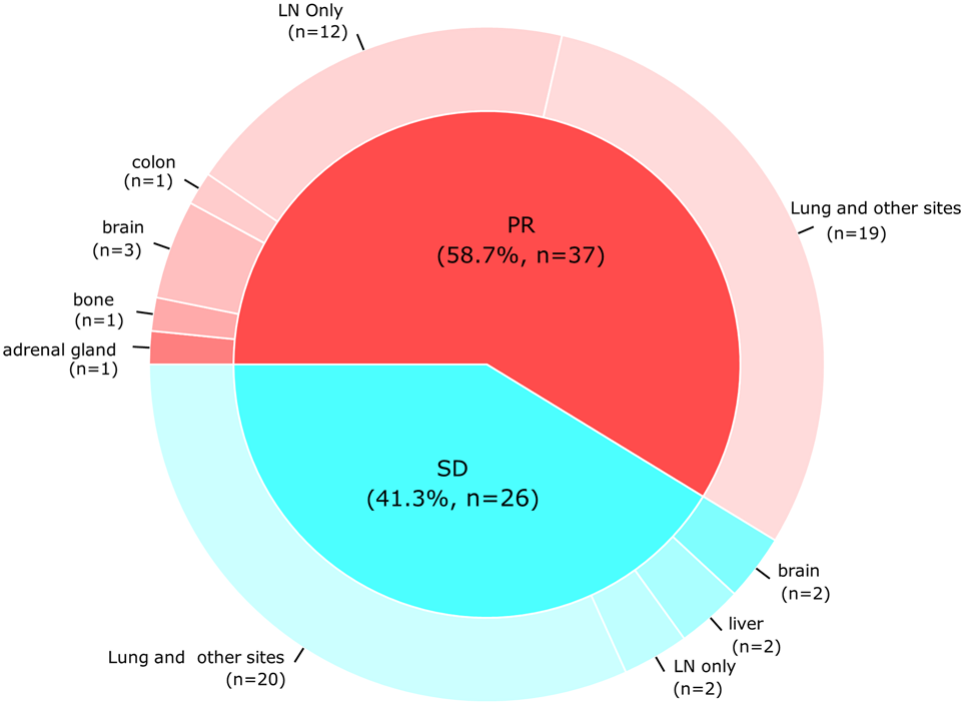
Donut plot showing sites of acquired resistance by best objective response to immune checkpoint inhibitors. *LN* lymph node, *PR* partial response, *SD* stable disease.

Among all the included patients, the median time to AR was 10.7 months. The clinical patterns of AR after ICI treatment are summarized in Table 2 and shown based on their best objective response in Fig. 1. The most common site of progressive disease was the lungs (38/63, 60.3%), followed by the LNs (31/63, 49.2%), and the brain (6/63, 9.5%). One of fifth (14/63, 22.2%) of patients had only LN progression at the time of AR. Twelve (85.7%) of the 14 patients with LN progression only had achieved PR as their best response, while 2 (14.3%) of the 14 patients achieved SD during ICI treatment (*P* = 0.04).

**Table 2:** Clinical Patterns of the failure of immune checkpoint inhibitors.

	All patients	1st/2nd line ICI	≥ 3rd line ICI	P-value
	(n = 63)	(n = 47)	(n = 16)	
**Previous best response**				
PR	37 (58.7)	16 (34.0)	6 (37.5)	0.089
SD (≥ 6 months)	26 (41.3)	31 (66.0)	10 (62.5)	
**Time to acquired resistance**				
Median (range)	10.7 (6.0–47.2)	10.8 (6.0–47.2)	10.2 (6.4–22.9)	0.052
**Overall survival after progression**				
Median (range)	10.9 (0.0–43.7)	11.5 (0.0–43.7)	9.6 (0.0–30.4)	0.775
**Number of progression sites**				
1	46 (73.0)	36 (76.6)	10 (62.5)	0.553
2	6 (9.5)	4 (8.5)	2 (12.5)	
≥ 3	11 (17.5)	7 (14.9)	4 (25.0)	
**Progression site**^**a**^				
Lung	38 (60.3)	23 (48.9)	15 (93.8)	**0.004**
Non-lung visceral organ				
Liver	4 (6.3)	2 (4.3)	2 (12.5)	1
Colon	1 (1.6)	1 (2.1)	0 (0)	
Adrenal gland	1 (1.6)	1 (2.1)	0 (0)	
Heart (pericardium)	2 (3.2)	1 (2.1)	1 (6.2)	
Brain	6 (9.5)	5 (10.6)	1 (6.2)	
Bone	4 (6.3)	2 (4.3)	2 (12.5)	1
Lymph node	31	24	7	0.265
Neck	5 (7.9)	3 (6.4)	2 (12.5)	0.274
Axilla	2 (3.2)	1 (2.1)	1 (6.2)	
Thoracic	20 (32.7)	16 (34.0)	4 (25.0)	
Abdomen	4 (6.3)	4 (6.3)	0 (0)	
**Pre-existence of progression site**				
New only	13 (20.6)	11 (23.4)	1 (6.2)	0.201
Existing only	43 (68.3)	32 (68.1)	12 (75.0)	
Both new and existing	7 (11.1)	4 (8.5)	3 (18.8)	
**Pattern of progression sites**				
Lymph node only	14 (22.2)	14 (30.0))	0 (0)	**0.013**
Extranodal organs	49 (77.8)	33 (70.0)	16 (100)	

Bold values denote statistical significance at the *p* < 0.05 level

*ICI* immune checkpoint inhibitors, *PR* partial response, *SD* stable disease.

^a^Patients were included in more than one group based on the site of progression.

Next, we evaluated the pattern of failure according to the type of ICI treatment. Less frequent lung progression was observed in patients treated with ICI as first- or second-line treatment than in patients treated with ICI as at least third-line treatment (48.9% vs. 93.8%; *P* value = 0.039). Progressive disease only in the LNs was observed among the patients who received first- or second line-treatment. There were no statistically significant differences between the two groups regarding the number of progression sites, previous best response, and the pre-existence of progression sites.

The clinical patterns of AR based on the number of progression sites are summarized in Table 3. Fifty-two (82.5%) patients had progression in one or two lesions (oligo-progression group), while 11 (25.4%) patients had progression in three or more lesions (multi-progression group). The lungs were the most frequent sites of progression in both groups. Progression of new lesions only was observed among 12 (23.1%) and 0 (0%) patients with oligo-progression and multi-progression, respectively (*P* < 0.001). Progression in the LNs only was also more frequent in patients with oligo-progression compared to patients with multi-progression (27.0% vs. 0%, *P* = 0.103).

**Table 3:** Baseline characteristics based on oligo-progression.

	Oligo-progression	Multiple progression	P-value
	(Progression site 1–2)	(Progression site 3 ≤)	
	(n = 52)	(n = 11)	
**Age (years)**			
Median (range)	64 (34–84)	62 (49–74)	0.752
**Sex**			
Male	38 (73.1)	9 (81.8)	0.714
Female	14 (26.9)	2 (18.2)	
**Smoking**			
Never smoked	19 (36.5)	2 (18.2)	0.31
Current or ex-smoker	33 (63.5)	9 (81.8)	
**Pathology**			
Squamous	16 (30.8)	3 (27.3)	0.822
Non-Squamous	36 (69.2)	8 (62.7)	
**ICI treatment line**			
01-Feb	40 (76.9)	7 (63.6)	0.449
3 ≤	12 (23.1)	4 (36.4)	
**Monotherapy**			
PD-1 inhibitor	27 (51.9)	9 (81.8)	
PD-L1 inhibitor	16 (30.8)	0 (0)	
**Combination therapy**			
PD-1 inhibitor + chemotherapy	1 (1.9)	0 (0)	
PD-1 inhibitor + CTLA-4 inhibitor	3 (5.8)	2 (18.2)	
PD-L1 inhibitor + CTLA-4 inhibitor	5 (9.6)	0 (0.0)	
**Previous response**			
SD	29 (55.8)	8 (72.7)	0.502
PR	23 (44.2)	3 (27.3)	
**Progression site**^**a**^			
Lung	28 (53.8)	10 (90.9)	**0.039**
Non-lung visceral organ			
Liver	2 (3.8)	2 (18.2)	0.275
Colon	1 (1.9)	0 (0)	
Adrenal gland	1 (1.9)	0 (0)	
Heart (pericardium)	1 (1.9)	1 (9.1)	
Brain	5 (9.6)	1 (9.1)	1
Bone	1 (1.9)	3 (27.3)	**0.015**
Lymph node	14 (23.6)	11 (100)	** < 0.001**
Neck	3 (5.8)	1 (9.1)	
Axilla	1 (1.9)	1 (9.1)	
Thoracic	10 (15.9)	10 (90.9)	
Abdomen	4 (6.3)	0 (0)	
**Pre-existence of progression site**			
New only	12 (23.1)	0 (0)	** < 0.001**
Existing only	39 (75.0)	5 (45.5)	
Both new and existing	1 (1.9)	6 (54.5)	
**Pattern of progression sites**			
Lymph node only	14 (27.0)	0 (0)	0.103
Extranodal organs	38 (73.0)	11 (100.0)	

Bold values denote statistical significance at the *p* < 0.05 level

*PR* partial response, *SD* stable disease, *PD-1* programmed death 1, *PD-L1* programmed death ligand 1, *CTLA-4* Cytotoxic T-Lymphocyte Associated Protein 4.

^a^Patients can be included in more than one group based on the site of progression^13^.

### Survival differences based on the progression patterns

**Figure 2:**
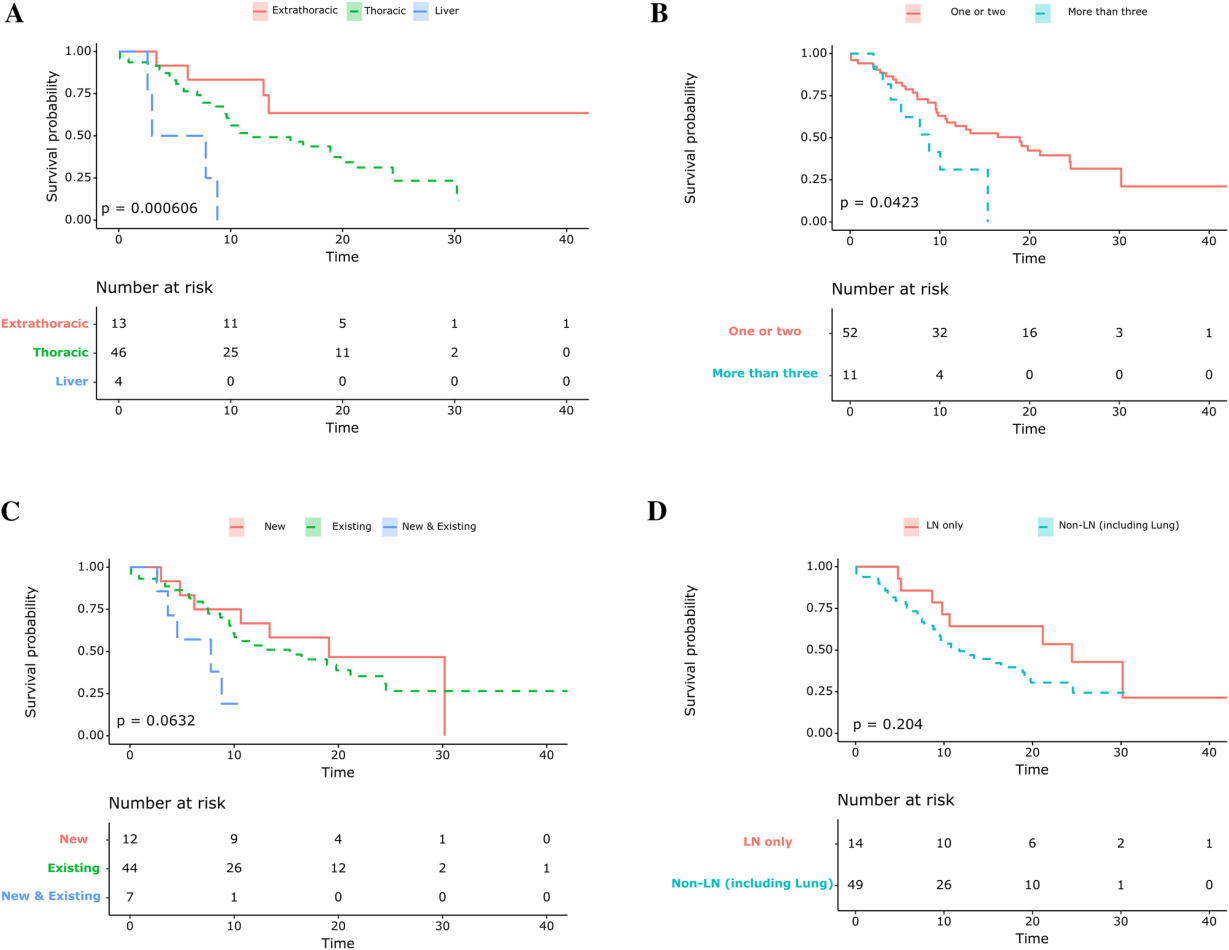
Kaplan–Meier survival curves showing overall survival from the point of acquired resistance for (**A**) the subgroup that had thoracic, extrathoracic, and liver progression only (**B**) the subgroup that had one or two progression sites or more than three progression sites (**C**) the subgroup that had progression from new lesions, existing lesions, and both new and existing lesions (**D**) the subgroup that had only lymph node and extranodal progression (including the lung).

The median OS after AR was 10.9 months. OS after AR was analyzed according to the pattern of progression. When the patients were classified into subgroups (i.e. those who had intrathoracic, extrathoracic [excluding the liver], and liver progression), the median OS after progression was significantly longer in the extrathoracic subgroup than in the thoracic and liver progression subgroups (30.2 months [95% CI, 13.4 to not reached (NR)], 11.7 months [95% CI, 9.5–21.1], and 5.4 months [95% CI, 2.6-NR], respectively, *P* < 0.001, Fig. 2A). The median OS after progression was significantly longer in the oligo-progression group compared to that in the multiple progression group (18.9 months [95% CI, 10.6-NR] vs. 8.8 months [95% CI, 5.7-NR], *P* = 0.04, Fig. 2B). The progression patterns were not statistically significant when classified by progression in new lesions (Fig. 2C) nor nodal progression (Fig. 2D).

### Subsequent therapy after AR

**Figure 3:**
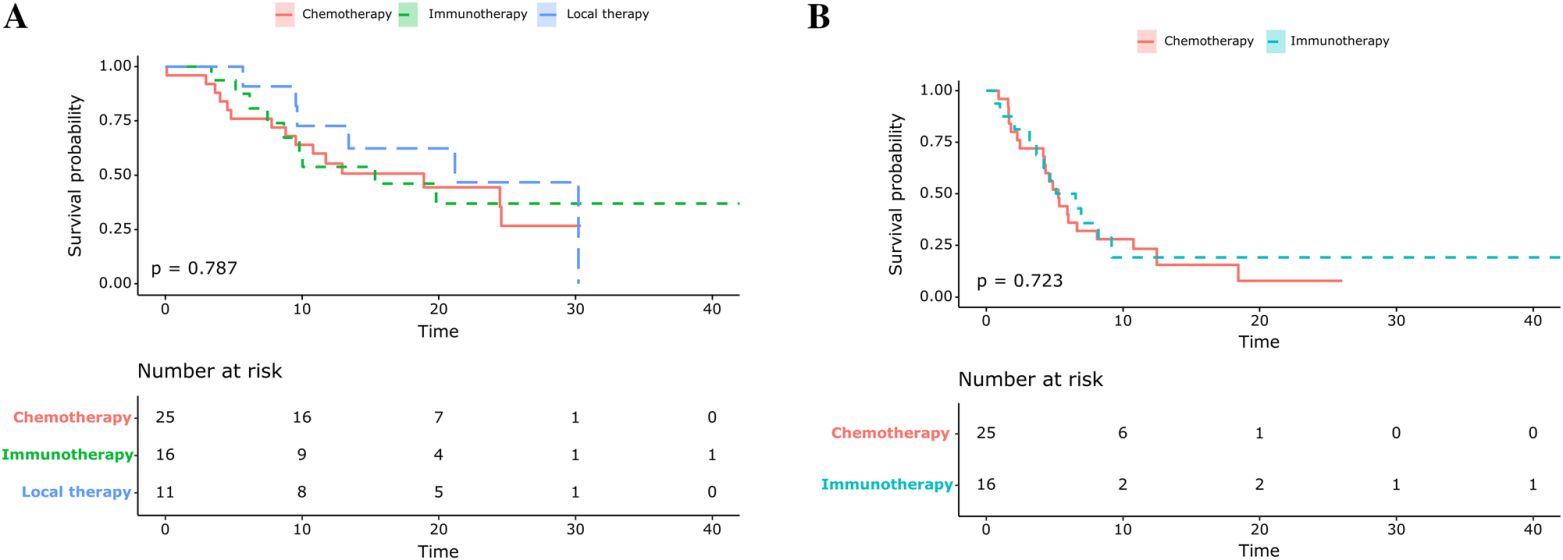
Kaplan–Meier survival curves showing (**A**) overall survival from the point of acquired resistance based on the subgroups of subsequent treatment (**B**) progression free survival based on the subsequent treatment.

Among the 63 patients with AR to ICIs, 52 (82.5%) patients received subsequent therapy after progression. Eleven (21.2%) patients received local treatment, including radiotherapy and surgical resection. Forty-one (78.8%) patients underwent systemic therapy after progression, including 25 (48.1%) and 16 (30.8%) patients who received cytotoxic chemotherapy and ICI after progression (Table 4). The median OS of the local therapy, chemotherapy, and ICI groups was 27.4, 23.2, and 29.1 months, respectively; there was no significant difference in survival between these groups (*P* = 0.787, Fig. 3A). Additionally, there was no significant difference in the PFS between the chemotherapy and the ICI groups (*P* = 0.723, Fig. 3B).

**Table 4:** Subsequent therapy after progression.

	All patients (n = 52)	1st/2nd line ICI (n = 40)	≥ 3rd line ICI (n = 12)
**Local therapy (n = 11)**			
Surgery	2 (3.8)	2 (5.0)	0 (0)
Radiotherapy	7 (13.5)	6 (15.0)	1 (8.3)
Gamma-knife surgery	2 (3.8)	2 (5.0))	0 (0)
**Systemic therapy (n = 41)**			
Immunotherapy	16 (30.8)	16 (40.0)	0 (0.0)
Chemotherapy	25 (48.1)	16 (40.0)	9 (75.0)

	All patients (n = 52)	Oligo-progression (Progression site 1–2) (n = 43)	Multiple progression (Progression site 3 ≤) (n = 9)
**Local therapy (n = 11)**			
Surgery	2 (3.8)	2 (4.7)	0 (0)
Radiotherapy	7 (13.5)	6 (14.0)	1 (11.1)
Gamma-knife surgery	2 (3.8)	2 (4.7)	0 (0)
**Systemic therapy (n = 41)**			
Immunotherapy	16 (30.8)	14 (32.6)	2 (22.2)
chemotherapy	25 (48.1)	19 (44.2)	6 (66.7)
**Local therapy (n = 11)**	Surgery	Colon mass excision (1) Axilla LN excision (1)	
	Radiotherapy	Abdomen LN (2)	
		Neck & SCN LN (1)	
		Lung mass (2)	
		Bone (2)	
	Gamma-knife surgery	Brain (2)	

*ICI* immune checkpoint inhibitors.

## Discussion

In this study, after a median time of 10.4 months, patients who had a durable clinical benefit experienced AR. The majority of these patients (52/63, 82.5%) had progressive disease in one or two sites. Progression in the LNs only was observed in 22.2% patients at the time of AR; such patients received ICI as first or second-line treatment. Most (12/14, 85.7%) patients with progression in the LNs only had objective tumor shrinkage, partial response according to RECIST, during ICI treatment. Among patients with oligo-progression (n = 52), progression of the LNs only (27.0%) or appearance of new lesions without evident progression of pre-existing lesions (23.1%) was observed. The patterns of progression observed in this cohort of patients with NSCLC at the time of AR are very unique considering that most patients treated in the conventional chemotherapy era develop systemic progression including pre-existing diseases. Even after having AR, patients with oligo-progression could be treated with local ablative therapy or continued ICI beyond AR. In the survival analysis, there was significantly longer OS among the oligo-progressors compared to that among systemic or multiple site progressors. We observed no significant difference in terms of PFS and OS between patients with subsequent chemotherapy and those with continuation of ICI beyond progression.

A notable finding in our study was that a significant portion of patients with AR to ICIs had progression of the LNs only, with sustained anti-cancer efficacy of ICI in pre-existing lesions. Among patients with a PR response with ICI (n = 37, 58.7%), 12 (32.4%) patients had progression of the LNs only. In the previous study by Gettinger et al. it was also reported that 42% of NSCLC patients who achieved PR after ICI treatment had AR limited to the LNs^11^. The study also suggested that the malignant LN environment may lead to the immunosuppressive evolution of cells within the node.

It should be also noted that that patients with oligo-progression showed prolonged OS compared to those with multi-progression after AR in our study. Potential hypotheses for this finding might be the “the cancer-immunity cycle”^15^. Tumors in still “immune surveillance active” hosts are not able to proliferate or metastasize to other organs unless they obtain the novel phenotype of immune evasion. Therefore, it is easily conceivable that lung cancer clones in the progressive site might have evolved to escape host immune surveillance with either loss of heterozygosity in human leukocyte antigens or depletion of the expressed neoantigens^16^.

In this study, physicians preferred local therapy and immunotherapy beyond progression for patients with oligo-progression. For NSCLC, there was an interesting retrospective report from the phase III OAK study that atezolizumab showed acceptable efficacy and safety after progression with patients with good performance^17^. To date, ICI has been reported to show post-progression efficacies in a limited subset of patients including those with metastatic urothelial carcinoma, renal cell carcinoma and melanoma, and NSCLC^18–22^. However, whether treatment beyond progression will be effective is not yet elucidated in patients with AR. Gettinger et al. reported that 11 NSCLC patients continued immunotherapy beyond AR^13^. Adam LC et al. reported an AR case of successful local control with cryotherapy combined with immunotherapy beyond progression^23^. Further analysis will help to define the clinical benefit for NSCLC patients with a durable response treated with ICI beyond progression.

There are several limitations to this study. First, this was a retrospective analysis. As not many patients showed a durable response to ICI, only a small number of patients were included in the study, resulting in limited statistical power. Therefore, the small study size prohibited us from performing comparisons between the different subgroups. Second, the study population was very diverse, including not only patients with PD-1 inhibitor or PD-L1 inhibitor monotherapy, but also patients with combination therapy including cytotoxic drugs or CTLA-4 inhibitors. Third, although the PD-L1 expression profile is a very important prognostic factor, our study included the PD-L1 profile utilizing three different anti-PD-L1 antibodies (22C3, E1L3N, and SP263). As most of the patients (81.0%) were involved in clinical trials, we were not able to ascertain the immunohistochemistry profiles for some patients, due to patient information protection regulations. Besides, information on co-medication such as antibiotics, corticosteroids, opioids, or proton pump inhibitors, which can affect ICI response, was not fully available. Lastly, there may be bias in the selection of subsequent therapy, as each individual physician was responsible for selecting the appropriate treatment.

Despite these limitations, our study described the clinical features and patterns of AR in detail. To our knowledge, this is the first study to show survival differences based on progression patterns. We believe that this report may provide useful information to clinicians regarding advanced NSCLC patients; additionally, it highlights the implications of subsequent therapy after AR.

In conclusion, advanced NSCLC patients who had durable responses (> 6 months) showed unique patterns of progression with high rates of progression among the LN only, in addition to oligo-progression. Local treatment and/or continuation of ICIs beyond AR might be an effective option for those patients. Further large-scale prospective studies are required to confirm these findings.

## Methods

### Design, patients, and data collection

We retrospectively reviewed the records of patients diagnosed with NSCLC and treated with inhibitors of PD-1 and PD-L1 at the Seoul National University Hospital (SNUH) and Seoul National University Bundang Hospital (SNUBH) between January 1, 2011 and November 31, 2018.

Adults without a previous history of severe systemic disease or autoimmune diseases who received PD-1/PD-L1 blockade alone or with cytotoxic T-lymphocyte associated protein 4 (CTLA-4) inhibitor or chemotherapy over the course of disease were included.

### Patient classification and outcome measures

The patients were classified into the following subgroups: those who had intrathoracic, extrathoracic [excluding the liver], and liver progression. AR after durable response was defined as disease progression after 6 months with the best response of the stable disease, partial response, or complete response according to the Response Evaluation Criteria in Solid Tumors (RECIST) v1.1 criterion. Imaging studies included Computed tomography scan and Magnetic resonance imaging and were independently reviewed by two observers. Progression-free survival (PFS) was calculated from the ICI treatment initiation date to the date of disease progression using the RECIST v1.1 criteria^24^, as confirmed by imaging, death, or the last follow-up date, if censored. OS was measured from the initiation of ICI treatment until death or the last follow-up date, if censored.

### Statistical analyses

Continuous variables were compared using the Students t-test or the Mann–Whitney U-test, whereas categorical variables were compared using the chi-squared or Fisher exact tests. Survival analyses were conducted according to the Kaplan–Meier method using the log-rank test. All tests were two-sided and *p*-values < 0.05 were considered to be statistically significant. For survival analysis, R version 3.4.3 software (R Development Core Team, https://www.r-project.org/) was used for computation.

### Ethics

All the included patients provided written, informed consent for data collection before treatment. The research team conducted retrospective analyses on cohort data. The institutional review boards of SNUH and SNUBH approved the study protocol (IRB number: B-2004-606-105). All procedures were carried out in accordance with the ethical standards of the institutional research ethics committee and the Helsinki declaration revised in 2013 by the World Medical Association.

## Supplementary Information

Supplementary Table 1.
