# Supplementary material for: Clinical pattern of failure after a durable response to immune check inhibitors in non-small cell lung cancer patients
Source: Sci Rep. 2021 Jan 28;11:2514. doi: 10.1038/s41598-021-81666-x (PMC7844257; doi:10.1038/s41598-021-81666-x)
Supplement: Supplementary file 1 — Supplementary Table 1. [file 41598_2021_81666_MOESM1_ESM.docx]

**Clinical pattern of failure after a durable response to immune check inhibitors in non-small cell lung cancer patients**

**Author Names**

Ja Yoon Heo^a§^, Shin Hye Yoo^a^, Koung Jin Suh ^b^, Se Hyun Kim^b*^, Yu Jung Kim^b^, Chan-Young Ock^a^, Miso Kim^a^, Bhumsuk Keam ^a,c^, Tae Min Kim ^a,c^, Dong-Wan Kim ^a,c^, Dae Seog Heo^a,c^ and Jong Seok Lee^b^

**Author Affiliations**

^a^Department of Internal Medicine, Seoul National University Hospital, Korea

^b^Department of Internal Medicine, Seoul National University Bundang Hospital, Korea

^c^Cancer Research Institute, Seoul National University, Seoul, Korea

§: The current affiliation is National Health Insurance Service Ilsan Hospital, Goyang, Korea.

**Corresponding author**

*Se Hyun Kim, MD, PhD

Department of Internal Medicine, Seoul National University

Bundang Hospital, Seoul National University College of Medicine, 82, Gumi-ro 173 beon-gil, Bundang-Gu, Seongnam, Gyeonggi-do 13620, Republic of Korea

E-mail: [sehyunkim@snubh.org](mailto:sehyunkim@snubh.org)

**Supplementary Table 1. Patient characteristics of acquired resistance**

| **Pt** | **ICI Line** | **ICI** | **Combination drug** | **ICI cycle** | **Best**  **response** | **irAE**  **(≥ grade 2 )** | **Imaging**  **Study** | **TTR** | **OS** | **re-biopsy** |
| --- | --- | --- | --- | --- | --- | --- | --- | --- | --- | --- |
| 01 | 1 | Nivolumab | - | 16 | PR | skin rash | CT | 8.3 | 33.4+ | No |
| 02 | 6 | Pembrolizumab | - | 11 | SD | - | CT | 8.1 | 32.7 | No |
| 03 | 4 | Pembrolizumab | - | 20 | SD | - | CT | 16.6 | 35.5 | No |
| 04 | 2 | Pembrolizumab | - | 65 | PR | - | CT | 47.2 | 55.9+ | Yes |
| 05 | 2 | Nivolumab | - | 17 | PR | - | CT | 8.6 | 33.0 | No |
| 06 | 1 | Nivolumab | - | 16 | SD | - | CT | 8.2 | 13.3+ | No |
| 07 | 2 | Pembrolizumab | - | 8 | PR | - | CT | 6.2 | 27.4 | No |
| 08 | 1 | Nivolumab | - | 91 | PR | Thyroiditis, Hepatitis | CT | 40.0 | 50.6+ | No |
| 09 | 3 | Durvalumab | - | 10 | SD | - | CT | 10.2 | 19.7 | No |
| 10 | 2 | Nivolumab | - | 20 | PR | Pneumonitis | CT | 12.8 | 25.7 | No |
| 11 | 2 | Atezolizumab | - | 14 | SD | - | CT | 10.3 | 30.1 | No |
| 12 | 2 | Atezolizumab | - | 44 | PR | - | CT,MR | 31.2 | 54.2+ | No |
| 13 | 2 | Atezolizumab | - | 29 | SD | - | CT | 20.7 | 49.7+ | Yes |
| 14 | 2 | Atezolizumab | - | 27 | PR | - | CT | 19.4 | 47.2+ | Yes |
| 15 | 1 | Durvalumab | - | 12 | SD | - | CT,MR | 12.0 | 15.4 | No |
| 16 | 2 | Avelumab | - | 21 | PR | Hypothyroidism | CT | 21.5 | 35.9+ | Yes |
| 17 | 1 | Nivolumab  +Ipilimumab | **-** | 10 | SD | - | CT | 6.0 | 13.2 | No |
| 18 | 4 | Nivolumab | - | 14 | SD | - | CT | 7.3 | 13.0 | No |
| 19 | 2 | nivolumab | - | 4 | SD | Pneumonitis, TB | CT | 9.4 | 31.2+ | No |
| 20 | 1 | Durvalumab  +Tremelimumab | - | 20 | SD | - | CT,MR | 20.1 | 26.2 | No |
| 21 | 1 | Nivolumab | - | 34 | SD | Thyroiditis | CT | 17.5 | 34.9+ | Yes |
| 22 | 1 | Durvalumab | - | 19 | PR | - | CT | 19.1 | 39.6+ | Yes |
| 23 | 3 | Durvalumab | - | 6 | PR | - | CT | 6.4 | 16.0 | No |
| 24 | 3 | Nivolumab | - | 14 | SD | Pneumonitis | CT | 7.1 | 33.5+ | No |
| 25 | 2 | Avelumab | - | 8 | SD | - | CT | 8.2 | 34.8+ | No |
| 26 | 1 | Nivolumab  +Ipilimumab | - | 16 | PR | - | CT | 9.5 | 33.3+ | Yes |
| 27 | 2 | Avelumab | - | 8 | SD | - | CT | 8.1 | 12.1 | No |
| 28 | 1 | Nivolumab | - | 38 | PR | - | CT | 19.5 | 31.0+ | No |
| 29 | 1 | Nivolumab | - | 21 | SD | Thyroiditis | CT | 10.9 | 34.4+ | No |
| 30 | 1 | Nivolumab | Pemetrexed  carboplatin | 19 | SD | - | CT | 9.8 | 12.7 | No |
| 31 | 3 | Durvalumab  +Tremelimumab | - | 9 | SD | skin rash | CT | 9.0 | 20.7 | No |
| 32 | 6 | Pembrolizumab | - | 8 | SD | - | CT,MR | 6.4 | 15.2 | Yes |
| 33 | 2 | Durvalumab | - | 29 | SD | - | CT | 29.0 | 38.8 | Yes |
| 34 | 1 | Nivolumab  +Ipilimumab | - | 29 | PR | - | CT | 13.7 | 27.1+ | No |
| 35 | 1 | Nivolumab  +Ipilimumab | - | 28 | PR | - | CT | 12.9 | 27.2+ | Yes |
| 36 | 1 | Nivolumab  +Ipilimumab | - | 17 | PR | thyroiditis, TB | CT | 8.1 | 18.2 | Yes |
| 37 | 1 | Nivolumab  +Ipilimumab | - | 30 | PR | thyroiditis, arthritis | CT | 14.0 | 24.9+ | Yes |
| 38 | 2 | Nivolumab | - | 23 | PR | - | CT | 10.8 | 21.6 | No |
| 39 | 2 | Pembrolizumab | - | 17 | SD | - | CT | 11.7 | 16.6+ | No |
| 40 | 1 | Pembrolizumab | - | 10 | PR | - | CT | 7.0 | 10.6 | No |
| 41 | 1 | Pembrolizumab | - | 21 | PR | - | CT | 14.9 | 25.7+ | No |
| 42 | 1 | Nivolumab  +Ipilimumab | - | 2 | SD | Pneumonitis | CT | 7.6 | 7.6 | No |
| 43 | 2 | pembrolizumab | - | 11 | PR | - | CT | 8.1 | 38.3 | Yes |
| 44 | 1 | pembrolizumab | - | 24 | PR | - | CT | 16.6 | 60.3+ | No |
| 45 | 3 | pembrolizumab | - | 32 | PR | - | CT | 22.9 | 42.0 | Yes |
| 46 | 2 | nivolumab | - | 17 | PR | - | CT | 8.9 | 13.4 | Yes |
| 47 | 2 | nivolumab | - | 56 | PR | - | CT | 28.2 | 43.5 | Yes |
| 48 | 2 | nivolumab | - | 80 | PR | pneumonitis | CT | 40.4 | 45.2 | Yes |
| 49 | 4 | nivolumab | - | 26 | SD | - | CT | 13.4 | 29.8 | No |
| 50 | 2 | Durvalumab | - | 6 | PR | - | CT,MR | 6.0 | 17.7 | No |
| 51 | 1 | Durvalumab | - | 9 | PR | - | CT | 9.5 | 16.5 | No |
| 52 | 2 | Durvalumab | - | 8 | PR | hypothyroidism, thrombocytopenia | CT | 8.3 | 14.1 | No |
| 53 | 5 | Durvalumab | - | 11 | PR | - | CT | 11.5 | 19.0 | No |
| 54 | 2 | Durvalumab  +tremelimumab | - | 10 | PR | - | CT,MR | 10.2 | 28.1+ | Yes |
| 55 | 2 | nivolumab | - | 13 | SD | neutropenic fever | CT | 6.7 | 9.2 | No |
| 56 | 5 | nivolumab | - | 24 | SD |  | CT | 12.3 | 12.3 | No |
| 57 | 4 | nivolumab | - | 19 | PR | - | CT | 9.4 | 18.9 | No |
| 58 | 2 | nivolumab | - | 15 | PR | - | CT | 7.8 | 25.1+ | Yes |
| 59 | 2 | nivolumab | - | 47 | PR | - | CT | 23.9 | 30.1+ | Yes |
| 60 | 4 | pembrolizumab | - | 18 | SD | - | CT | 13.2 | 43.6+ | Yes |
| 61 | 4 | nivolumab | - | 33 | PR | adrenal insufficiency, pneumonitis | CT | 16.6 | 19.2 | Yes |
| 62 | 2 | nivolumab | - | 21 | PR | - | CT | 10.7 | 11.6 | No |
| 63 | 4 | Nivolumab | - | 20 | PR | colitis | CT | 10.3 | 18.0 | Yes |

Pt, patient; ICI, immune checkpoint inhibitors; irAE, immune-related adverse event; PR, partial response; SD, stable disease; CT, Computed tomography; MR, Magnetic resonance imaging; TTR, time to acquired resistance; OS, overall survival;
